# Artificial intelligence accuracy in detecting pathological breath sounds in children using digital stethoscopes

**DOI:** 10.1186/s12931-020-01523-9

**Published:** 2020-09-29

**Authors:** Ajay Kevat, Anaath Kalirajah, Robert Roseby

**Affiliations:** 1 Department of Paediatrics, Monash University, Melbourne, Australia; 2 Department of Respiratory Medicine, Monash Children’s Hospital, 246 Clayton Road, Clayton, Melbourne, Victoria 3168 Australia

**Keywords:** Artificial intelligence, Auscultation, Child, Respiratory sounds, Stethoscopes

## Abstract

**Background:**

Manual auscultation to detect abnormal breath sounds has poor inter-observer reliability. Digital stethoscopes with artificial intelligence (AI) could improve reliable detection of these sounds. We aimed to independently test the abilities of AI developed for this purpose.

**Methods:**

One hundred and ninety two auscultation recordings collected from children using two different digital stethoscopes (Clinicloud™ and Littman™) were each tagged as containing wheezes, crackles or neither by a pediatric respiratory physician, based on audio playback and careful spectrogram and waveform analysis, with a subset validated by a blinded second clinician. These recordings were submitted for analysis by a blinded AI algorithm (StethoMe AI) specifically trained to detect pathologic pediatric breath sounds.

**Results:**

With optimized AI detection thresholds, crackle detection positive percent agreement (PPA) was 0.95 and negative percent agreement (NPA) was 0.99 for Clinicloud recordings; for Littman-collected sounds PPA was 0.82 and NPA was 0.96. Wheeze detection PPA and NPA were 0.90 and 0.97 respectively (Clinicloud auscultation), with PPA 0.80 and NPA 0.95 for Littman recordings.

**Conclusions:**

AI can detect crackles and wheeze with a reasonably high degree of accuracy from breath sounds obtained from different digital stethoscope devices, although some device-dependent differences do exist.

## Background

Accurately detecting abnormal breath sounds is vital in clinical pediatric medicine, as the nature and presence of pathological sounds guides diagnosis and initial treatment of common respiratory conditions. However, use of a standard binaural stethoscope by human practitioners to detect abnormal chest sounds introduces assessment subjectivity and research has shown that significant inter-listener variability exists [1–3]. This calls into question the accuracy of diagnoses made on the basis of human auscultation. Treatment decisions informed by the diagnosis made may therefore be misguided, leading to unnecessary side effects and delay in provision of effective treatment.

In recent years, stethoscopes capable of digitally recording breath sounds have become more widely available, offering the ability to capture breath sounds with superior sound quality and fidelity [4]. However, human interpretation of the digital recordings can still exhibit significant inter-listener variability [5]. As the soundwave properties of pathologic breath sounds such as crackles, wheezes and rhonchi have been well-studied and previously defined, computer algorithms and programs to automatically detect them have been developed [6, 7]. Increasingly, artificial intelligence (AI) algorithms have been applied in medicine, and because they have the capability of self-improvement as they learn from new data and cases, they can evolve to outperform traditional signal processing techniques [8, 9]. AI programs based upon neural network programming have been used to identify melanomas from photographs of skin and suspicious soft tissue / calcified lesions on routine mammograms with an accuracy greater than most dermatologists and radiologists respectively, who were interpreting those images for performance comparison [10, 11]. Similarly, an AI algorithm designed to detect abnormal pediatric breath sounds based upon several thousand patient recordings collected using a digital stethoscope (DS) was reported to outperform pediatricians, especially in coarse crackle detection [12]. However, the study, which was primarily conducted by developers of the technology, utilized breath sounds collected using only the StethoMe DS. Therefore, we aimed to establish the performance of the AI algorithm in detecting pathological pediatric breath sounds collected using other DS devices, to evaluate the algorithm’s generalizability. We used blinding techniques and real-world recordings to maximize the validity and applicability of our study.

## Methods

**Fig. 1:**
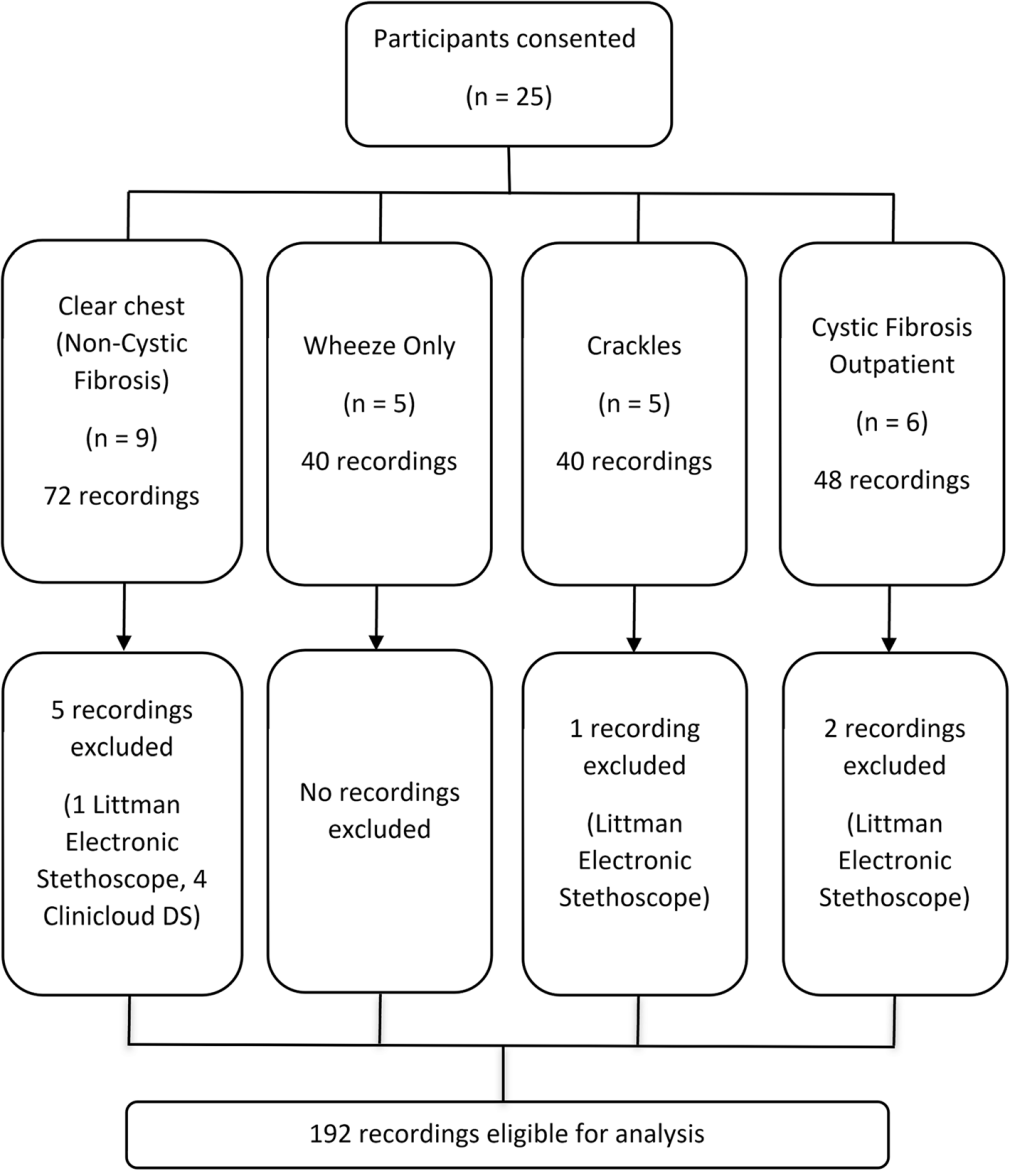
Participant recruitment and recording analysis flowchart

We obtained breath sound recordings from a sample of twenty-five pediatric patients from four groups (Fig. 1) at Monash Children’s Hospital, a tertiary institution in Melbourne, Australia. Eligible patients and carers were sequentially approached during times in which the study’s data collector was available. Written informed consent and human research ethics committee approval (reference number 15327 L) was obtained. We excluded those receiving oxygen or positive pressure ventilation. Participants were grouped by auscultation findings determined by their attending senior pediatric doctor using a standard binaural stethoscope: group 1, normal breath sounds (*n* = 9); group 2, wheeze only (*n* = 5); group 3, crackles with or without wheeze (n = 5) and group 4, cystic fibrosis (CF) clinic attendees with a clear chest according to their respiratory physician (*n* = 6).

Recordings were obtained in the clinical setting, with the child’s upper body clothing removed. Immediately after standard auscultation, a twenty-second recording from each quadrant of the posterior thorax was taken, using a Clinicloud™ DS and a Littman™ 3200 Electronic Stethoscope. Recordings were downloaded onto a computer and processed with normalization and bandpass filtering (high-pass 6th order Butterworth filter at 100 Hz plus low-pass 4th order Butterworth filter at 1000 Hz) to minimize heartbeat and extraneous sound intrusion. Four recordings obtained by each DS were excluded due to aberrant recording, occurring due to inadequate chest wall contact made with the stethoscope head at the time of the recording and/or cable connection failure. Using combined spectrographic, sound wave and audio playback analysis by a pediatric respiratory physician as previously described [13], recordings were carefully checked and tagged for the presence or absence of crackles and wheezes/rhonchi. As characteristic audiologic morphologies of crackles and wheezes/rhonchi have been previously elucidated, we used the following cited established descriptions to define and identify crackles and wheezes/rhonchi in the DS recordings on an a priori basis: crackles – a short initial soundwave deflection from a baseline followed by a longer, dampening sinusoidal wave with < 20 msec two-cycle duration and < 25 msec total duration width [14], and wheeze/rhonchi - a rapid periodic sinusoidal waveform of total length > 25 msec with a dominant frequency > 100 Hz [6]. To verify accuracy of tagging, recordings from 20% of participants were analyzed by a blinded second clinician in the same fashion.

Untagged versions of the recordings in wavefile (.wav) format devoid of participant information were analyzed using StethoMe AI, a neural network based AI algorithm that provided numerical scores for each recording, with a separate score each for crackles and wheeze/rhonchi. Each generated score, known as the probability raster, represented the likelihood of presence of crackles or wheeze/rhonchi detected by the automated system. The neural network AI was trained and validated on a set of > 10,000 real (not synthetically-generated) recordings that did not include the recordings collected in this study. Details of the specialized neural network and its probability raster output have been previously described [12]. Persons handling the AI and the AI itself were blinded to tagged recordings as they were sent only untagged versions, and no further recording exclusion due to the AI algorithm perceiving poor audio quality (or for any other reason) was permitted.

Neural network based pathological breath sound detection was compared with tagged outcomes on a per-recording basis. Positive percent agreement (PPA), true positive rate (TPR) and negative percent agreement (NPA) values were generated for different threshold cutoffs applied to the numerical probability raster scores, with a receiver operating characteristic (ROC) curve used to identify optimal cutoffs that represented best neural network performance. Due consideration was given to achieving a balance between PPA, which is similar to sensitivity, and NPA, which is similar to specificity. This analysis was performed for each pathological breath sound type (wheeze/rhonchi, and crackles). Analysis was conducted for each DS type (Clinicloud, Littman) separately.

## Results

We studied 192 recordings from 25 children with a median age of 6.7 years and median weight of 22.2 kg (Table 1). The Cohen’s kappa assessing inter-rater agreement for scoring/tagging of the subset of recordings analyzed by the blinded second clinician was 1.0. Per recording, the AI algorithm was able to deliver two probability raster scores (one for crackles and one for wheeze/rhonchi) for 100% of submitted recordings. Scores ranged from 0.00 (AI suggesting absence of the pathologic breath sound) to 0.88 (AI suggesting very high probability of present pathologic breath sound). Using a probability raster cutoff of > 0.00 to maximize sensitivity in detecting abnormal breath sounds resulted in a TPR of 0.95 (PPA 0.86, NPA 0.99) for crackles from Clinicloud recordings and a TPR of 0.75 (PPA 0.6, NPA 0.96) for crackles from Littman recordings. For wheeze detection using the same cutoff, TPR was 0.93 (PPA 0.90, NPA 0.97) for Clinicloud recordings and 0.8 (PPA 0.76, NPA 0.95) for Littman recordings.

**Table 1:** Patient Characteristics

Demographics	N (% or interquartile range)
Female	7 (28)
Age, median (years)	6.7 (3.4)
Weight, median (kilograms)	22.2 (11.4)
Patient Groups	
Patients with cystic fibrosis (CF)	6 (24)
Normal breath sounds, without CF	9 (36)
Wheeze only^a^	5 (20)
Crackles ± wheeze^a^	5 (20)

^a^patients in these groups were diagnosed with lower respiratory tract infection, asthma or preschool wheeze

**Fig. 2:**
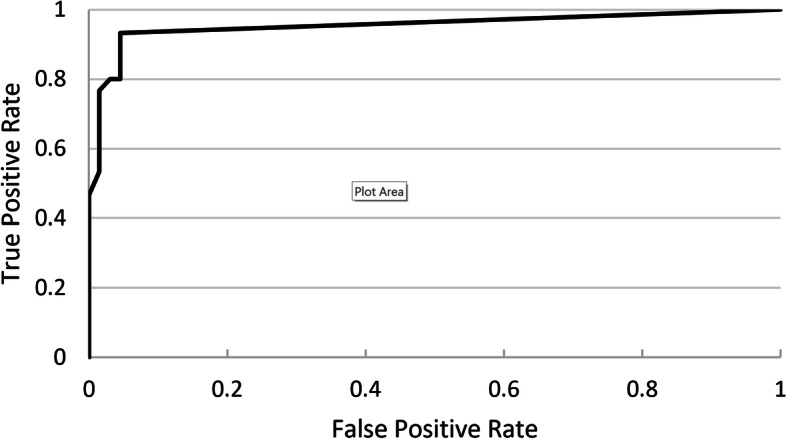
Receiver Operating Characteristic (ROC) Curve: AI performance in detecting. Clinicloud-recorded wheeze

**Fig. 3:**
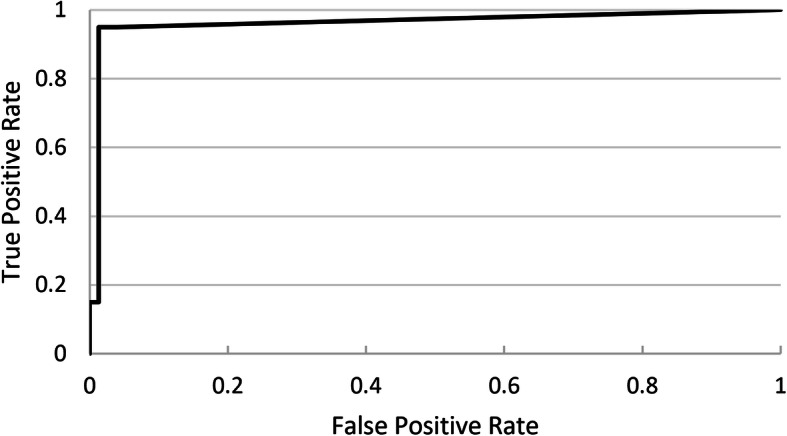
Receiver Operating Characteristic (ROC) Curve: AI performance in detecting. Clinicloud-recorded crackles

**Fig. 4:**
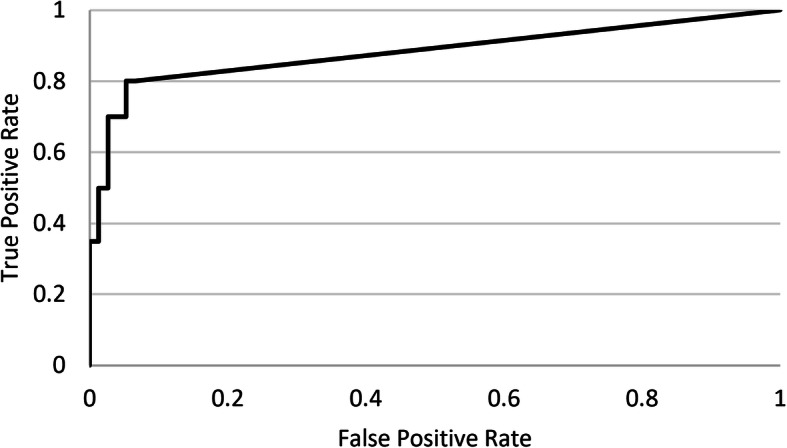
Receiver Operating Characteristic (ROC) Curve: AI performance in detecting. Littman-recorded wheeze

**Fig. 5:**
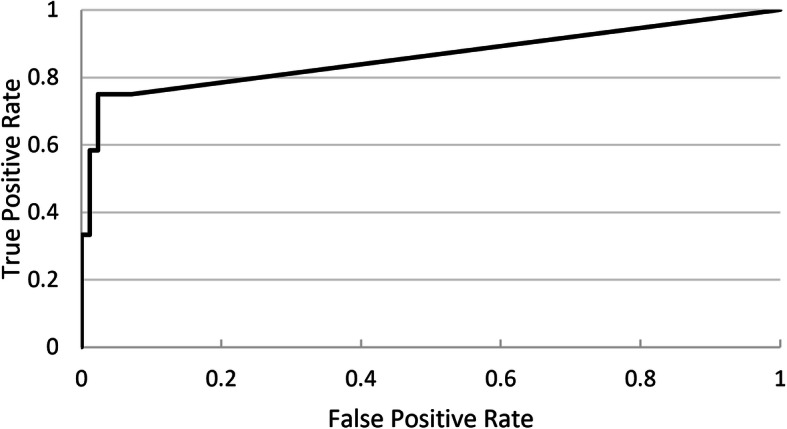
Receiver Operating Characteristic (ROC) Curve: AI performance in detecting. Littman-recorded crackles

Ideal probability raster cutoffs (i.e. score above which recordings are considered positive for the presence of crackles or wheeze/rhonchi) were determined by construction of ROC curves (Figs. 2, 3, 4 and 5). For Clinicloud-based crackle detection, an optimized cutoff score of anywhere between 0.029 and 0.037 yielded a PPA of 0.95 (NPA 0.99), whereas for Littman-based crackle identification, using any cutoff from 0.024 to 0.03 resulted in a PPA of 0.82 (NPA 0.96). For wheeze detection, utilizing a probability raster cutoff score between 0.001 and 0.005 returned a PPA of 0.90 (NPA 0.97) for Clinicloud recordings, whereas for wheeze identification from Littman recordings, an optimized cutoff score of between 0.004 and 0.006 had a PPA of 0.80 (NPA 0.95).

## Discussion

Our independent, blinded validation of an existing AI using recordings from foreign DS devices shows the technology has the ability to detect abnormal pediatric breath sounds with a promising level of accuracy similar to or better than its performance when tested on recordings from the DS it was originally trained with [12]. This demonstrates generalizability of the AI solution.

As there is no suitable ‘gold standard’ benchmark for identifying the presence or absence abnormal breath sounds, we used careful audio and spectrogram analysis of real-world, in-hospital recordings to determine this, with validation by a second blinded clinician who applied the same strict labelling criteria demonstrating excellent interrater agreement. Although human expert labelling may introduce potential bias, previous multicenter research has shown that this approach is a method suitable for research of lung sounds [5]. Nevertheless, due to the absence of a true gold standard, we report our results using PPA and NPA, rather than the more familiar terms sensitivity and specificity.

Our study has some limitations. Firstly, our pediatric test data was collected using only two different DS devices, whereas several more are now available [4]. We chose these two devices due to either their popularity or local availability. Although optimal algorithmic threshold cutoffs to maximize AI accuracy were similar and overlapping for the two devices tested, it is unclear if subtle device-dependent differences regarding the optimal AI cutoff settings exist. Furthermore, the AI performed better using recordings from one DS compared to the other. We speculate this to be due to differences in the audio quality of the recordings outputted by each DS; the Littman Electronic Stethoscope outputted files with a sample rate of 4000 Hz (16-bit) whereas the Clinicloud DS did so at 44,100 Hz (32-bit), producing a noticeable resolution difference when viewed spectrographically [13].

Secondly, the size of our recording set, whilst suitable for the purpose of initial independent testing of the AI, was not large enough to test the neural network’s ability to improve further with additional training. Finally, we were not able to directly compare the performance of the AI algorithm against the judgement of treating pediatric clinicians, as clinician opinion on the presence or absence of abnormal breath sounds per recording area / quadrant auscultated was not obtained. We do note however that when this has previously been performed, the neural network classification often more closely matched the established standard than clinician judgement [15].

## Conclusion

In recent years, AI algorithms have been developed that perform as well or better than expert humans across a range of specific tasks both within and outside of clinical respiratory medicine [9]. As the integration of AI into medical care gains momentum, independent validation of AI capabilities and weaknesses is important to undertake in order to ensure quality control [16]. Because there are significant ethical, legal and social implications inherently tied to the way in which medical decisions are made, we have a duty to ensure that making changes to this process, for example by incorporating machine judgement, is indeed a beneficial step [8].

Our study independently validated the performance and generalizability of an AI algorithm (StethoMe AI) for the detection of pathological breath sounds in DS recordings obtained from a population of children with and without respiratory illness. We demonstrated the neural network could identify pediatric breath sounds with similar capability across multiple DS devices, although some device-dependent differences do exist. Accuracy of the AI solution is promising and is at least similar to that of many clinicians routinely assessing and guiding medical decisions for these children in current clinical practice. Future efforts should focus upon improving diagnostic accuracy, developing algorithm elements that can reliably track and predict patient improvement and/or deterioration over time, and safely assessing potential impacts of AI use in real-world clinical environments.

## Data Availability

The datasets generated during and/or analyzed during the current study are available from the corresponding author on reasonable request.

## Abbreviations

AI: Artificial intelligence
CF: Cystic fibrosis
DS: Digital stethoscope
Hz: Hertz
msec: Millisecond
NPA: Negative percent agreement
PPA: Positive percent agreement
ROC: Receiving operator characteristic
TPR: True positive rate

## References

[1] Wipf JE, Lipsky BA, Hirschmann JV, Boyko EJ, Takasugi J, Peugeot RL (1999). Diagnosing pneumonia by physical examination. Arch Intern Med.

[2] Brooks D, Thomas J (1995). Interrater reliability of auscultation of breath sounds among physical therapists. Phys Ther.

[3] Prodhan P, Dela Rosa RS, Shubina M, Haver KE, Matthews BD, Buck S (2008). Wheeze detection in the pediatric intensive care unit: comparison among physician, nurses, respiratory therapists, and a computerized respiratory sound monitor. Respir Care.

[4] Ramanathan A, Zhou L, Marzbanrad F, Roseby R, Tan K, Kevat A (2019). Digital stethoscopes in paediatric medicine. Acta Paediatr.

[5] Aviles-Solis JC, Vanbelle S, Halvorsen PA, Francis N, Cals JWL, Andreeva EA (2017). International perception of lung sounds: a comparison of classification across some European borders. BMJ Open Respir Res.

[6] Gurung A, Scrafford CG, Tielsch JM, Levine OS, Checkley W (2011). Computerized lung sound analysis as diagnostic aid for the detection of abnormal lung sounds: a systematic review and meta-analysis. Respir Med.

[7] Reichert S, Gass R, Brandt C, Andrès E (2008). Analysis of respiratory sounds: state of the art. Clin Med Circ Respirat Pulm Med.

[8] The Lancet (2017). Artificial intelligence in health care: within touching distance. Lancet..

[9] Gonem S, Jannsens W, Das N, Topalovic M (2020). Applications of artificial intelligence and machine learning in respiratory medicine. Thorax..

[10] Esteva A, Kuprel B, Novoa RA, Ko J, Swetter SM, Blau HM (2017). Dermatologist-level classification of skin cancer with deep neural networks. Nature..

[11] Rodriguez-Ruiz A, Lång K, Gubern-Merida A, Broeders M, Gennaro G, Clauser P (2019). Stand-alone artificial intelligence for breast cancer detection in mammography: comparison with 101 radiologists. J Natl Cancer Inst.

[12] Grzywalski T, Piecuch M, Szajek M, Bręborowicz A, Hafke-Dys H, Kociński J (2019). Practical implementation of artificial intelligence algorithms in pulmonary auscultation examination. Eur J Pediatr.

[13] Kevat AC, Kalirajah A, Roseby R (2017). Digital stethoscopes compared to standard auscultation for detecting abnormal paediatric breath sounds. Eur J Pediatr.

[14] Sarkar M, Madabhavi I, Niranjan N, Dogra M (2015). Auscultation of the respiratory system. Ann Thorac Med.

[15] Grzywalski T, Szajek M, Hafke-Dys H, Bręborowicz A, Kociński J, Pastusiak A (2019). Respiratory system auscultation using machine learning - a big step towards objectivisation? [abstract]. Eur Respir J.

[16] Challen R, Denny J, Pitt M, Gompels L, Edwards T, Tsaneva-Atanasova K (2019). Artificial intelligence, bias and clinical safety. BMJ Qual Saf.

